# Treatment of Fresh, Open Wounds

**Published:** 1885-02-20

**Authors:** Q. C. Smith

**Affiliations:** Austin, Texas


### TREATMENT OF FRESH, OPEN WOUNDS.

By Q. C. SMITH, M. D., Austin, Texas.

It is presumed that every physician has made himself familiar with the various modes of treatment of fresh, open wounds, as set forth in modern standard works on surgery, therefore we forbear to consume your time or insult your intelligence by going through a tedious rehearsal of the history of this subject.

We do not pretend to anything new or original, but believe it may be profitable, at least to some, to remind them of certain means and measures that experience has demonstrated as being highly conducive to the rapid healing of fresh, open wounds in a safe and comfortable manner.

The general method and principles of treating fresh, open wounds—whether they be incised, lacerated, contused, or punctured—which we advocate will apply to either class, with such variation as the severity or class of wound would indicate, the same general principles being observed in all cases. If the wound be comparatively severe, the limb or part wounded should be so placed as to most favor free arterial circulation through the part near around the wound, and a free flow of the venous blood away from the wound.

---

*To be strictly accurate, the property of alcohol as a *paralyzant* was at first pointed out by Dr. T. W. Poole, of Ontario, in a book published by him in 1879. Dr. C. H. Hughes has also several times called attention to the same point.

To facilitate brevity and precision, we will now state the composition of two lotions we use in dressing fresh, open wounds :

The first is made by adding compound tincture of iodine to clean hot water until the water is light cherry-red ; this we will designate the "cleansing lotion," which should be used as hot as is comfortable to the hand of the operator. The second lotion, which we call the "healing lotion," is made thus :

℞. Spts. turpentine.......... ℥ ii,

Gum acacia.......... ʒ i,

Aqua dist.......... ℥ ii,

Mix well. Add :

Camphorated tinct. opii.........
Pure white sugar, in fine powder } aa............ ℥ ii.

Mix well.

Of course, in dressing fresh, open wounds, bleeding must be arrested by ligating the larger vessels, if necessary. Then the cleansing lotion should be freely streamed over the surface of the wound with a clean spongue, using the lotion hot, pure, and clean, until all blood-oozing has entirely ceased, and for several minutes longer. During the cleansing process the raw surfaces should not be wiped or even touched with the sponge.

As soon as the cleansing lotion has ceased to drop from the wound, the healing lotion should be freely and thoroughly applied to every part of the wound.

The wound is now ready to be coaptated, which should be done in a gentle, accurate manner, using as few sutures as possible, using preferably carefully graduated, even-pressure by bandages over absorbent cotton compresses. A good sized pledget of absorbent cotton—proportioned to the size of the wound—saturated with the healing lotion, should be snugly placed over the joined edges of the wound, and over this a thick compress of dry, absorbent cotton, held in place by carefully applied bandages, for gentle, steady compression is a great aid to quick union, and a powerful safeguard against septicæmia. If the wound is of such shape, or condition, that it cannot be coaptated, the foregoing mode of dressing will still be in order, the absorbent cotton pledget saturated with the healing lotion, to be closely applied to all parts of the raw surface, and well covered with dry absorbent cotton.

Fresh wounds that can be accurately coaptated, dressed in this manner, usually heal by first intention, and even extensive lacerated contused wounds will rarely suppurate, but heal rapidly, giving little pain, never producing septicæmia.

Wounds thus dressed should not be molested as long as they are comfortable and emit no odor, or show no sign of suppuration. As a rule, with rare exceptions, wounds thus dressed should not meet with water after the primary dressing, as dryness is conducive to safe and rapid healing. In subsequent dressings the same means and principles before referred to should be carefully observed.

But should the healing process cease to progress, the healing lotion should be withheld, and, for a few days at least, the wound dressed with the following powder :

℞. Socot aloes . . }
Iodoform . . . . } aa, in fine powder . . . . . . . . . . . . . . . ℥ i,
Boraccic acid, }
Acetate morphia, . . . . . . . . . . . . . . . . . . . . . . . . . . . . . gr. x,
Subnit. bismuth, q. s. ad . . . . . . . . . . . . . . . . . . . . . . . . ℥ ii.
M. Pt. pow.

S. : Spread freely over all the raw surface of the wound, and renew the dressing; soon as the wound discharges, begin to moisten the dressing, or the wound emits a perceptible odor.

Wounds dressed after this plan, however extensive, if properly attended to, very rarely emit any odor whatever.

Last, but by no means least, the surgeon should not for a moment forget that his wounded patient is a sick man, and begin at once, without delay, to use all appropriate means, hygienic, pharmaceutic, and psychical, calculated to cause the constructive machinery of his life to perform its every function to the highest possible degree of perfection.

As we stated in the outset, we have not presumed to tell anything new, for the mendicants we recommend have been in use more than a thousand years ; or labored to achieve originality, but simply to remind you of some old, good things, appertaining to the treatment of fresh, open wounds, which we can confidently assure you are at least equal in efficiency in every respect—and we know whereof we speak—and far more safe, simple and convenient than the elaborate toilet of the modern carbolic acid, so-called antiseptic treatment.

*En passant*, we would remark that our so-called healing lotion is a most admirable dressing for burns and scalds.—*Trans. Texas Med. Association*, *1884*.

